# Computational insights into fluconazole resistance by the suspected mutations in lanosterol 14α-demethylase (Erg11p) of *Candida albicans*

**DOI:** 10.22099/mbrc.2020.36298.1476

**Published:** 2020-12

**Authors:** Sagunthala Murugesan Udaya Prakash, Yasin Nazeer, Sivaraman Jayanthi, Mohammad Anaul Kabir

**Affiliations:** 1 Molecular Genetics Laboratory, School of Biotechnology, National Institute of Technology Calicut, Calicut 673601, Kerala, India; 2 Regional Medical Research Centre, Indian Council of Medical Research (ICMR), Belagavi -590010, Karnataka, India; 3 Computational Drug Design Lab, School of BioSciences and Technology, Vellore Institute of Technology, Vellore-632014, Tamil Nadu, India

**Keywords:** Candida albicans, Drug resistance, Suspected mutations, Molecular docking

## Abstract

Mutations in the ergosterol biosynthesis gene 11 (*ERG11*) of *Candida albicans* have been frequently reported in fluconazole-resistant clinical isolates. Exploring the mutations and their effect could provide new insights into the underlying mechanism of fluconazole resistance. Erg11p_Threonine285Alanine (Erg11p_THR285ALA), Erg11p_Leucine321Phenylalanine (Erg11p_LEU321PHE) and Erg11p_Serine457Proline (Erg11p_SER457PRO) are three fluconazole-resistant suspected mutations reported in clinical isolates of *C. albicans*. Therefore, our study aims to investigate the role of these suspected mutations in fluconazole resistance using in-silico methods. Molecular dynamics simulation (MDS) analysis of apo-protein for 25ns (nanosecond) showed that suspected mutant proteins underwent slight conformational changes in the tertiary structure. Molecular docking with fluconazole followed by binding free energy analysis showed reduced non-bonded interactions with loss of heme interaction and the least binding affinity for Erg11p_SER457PRO mutation. MDS of suspected mutant proteins-fluconazole complexes for 50ns revealed that Erg11p_SER457PRO and Erg11p_LEU321PHE have clear differences in the interaction pattern and loss or reduced heme interaction compared to wild type Erg11p-fluconazole complex. MDS and binding free energy analysis of Erg11p_SER457PRO-fluconazole complex showed the least binding similar to verified mutation Erg11p_TYR447HIS-fluconazole complex. Taken together, our study concludes that suspected mutation Erg11p_THR285ALA may not have any role whereas Erg11p_LEU321PHE could have a moderate role. However, Erg11p_SER457PRO mutation has a strong possibility to play an active role in fluconazole resistance of *C. albicans*.

## INTRODUCTION

Drug resistance acquired by opportunistic fungal pathogen *Candida albicans *is often observed in clinical isolates. Invasive infections in humans are widely caused by the drug-resistant *C. albicans*. This infection leads to high morbidity and mortality rate and reported to be the fourth most common nosocomial bloodstream infection [1-2]. Azole antifungals which were discovered around 40 years ago are the largest class of antifungal agents that are widely used in clinical practice. Among the azole drugs, fluconazole is commonly used as a first-line drug for the treatment of *Candida* infections due to its low toxic effect and tolerability [3]. The primary target of fluconazole is the inhibition of lanosterol 14α-demethylase which is encoded by the *ERG11* gene and is responsible for the synthesis of a major lipid component ergosterol in *C. albicans*. Inhibition of this enzyme leads to the depletion of cell membrane ergosterol which severely affects the integrity of the plasma membrane [4]. Subsequently, essential molecules leak out from *Candida* cells leading to its highly reduced growth or death. 

Drug resistance to fluconazole of *Candida* species has been increased in the past two decades. Fluconazole resistance of *C. albicans* was first reported in the late 1980s. According to a 2019 report by the Centers for Disease Control and Prevention (CDC), the United States of America, fluconazole-resistant *Candida* species accounts for 44,800 cases and 2,200 deaths. Studies have shown that multiple and diverse mechanisms including drug efflux pumps, overexpression of drug targets, target alterations and metabolic bypasses are responsible for drug resistance in *C. albicans*. Among these, drug target alterations due to the mutations in certain genes play a critical and significant role in developing antifungal drug resistance [4]. Importantly, point mutations in the *ERG11*gene of *C. albicans *are found to be associated with fluconazole resistance and the corresponding mutated lanosterol 14α-demethylase showed reduced binding affinity to fluconazole [5]. Approximately, 160 residue mutations have been reported in the Erg11 protein. However, only 10 of those mutations have been confirmed to be involved in fluconazole resistance [6]. 

In the modern era, in-silico techniques play a very important role in the biological system worldwide. In-silico mutation techniques are used to develop mutations in protein crystal structure to have insights into the conformational changes and stability of the proteins [7]. Currently, in-silico techniques are widely used for predicting various biological mechanisms like drug sensitivity and resistance, protein-protein interactions and DNA-protein interactions. The amino acid substitutions in Erg11 protein is one of the most important mechanisms contributing to azole resistance in *C. albicans* [8]. The study of residue mutations in Erg11p will help us in identifying the crucial amino acids that may involve in drug resistance of *C. albicans*. 

As mentioned above, in-silico techniques would be of significant importance in exploring residue mutations involved in drug resistance before proceeding for rigorous wet lab experiments. Crystal structures available in the Protein Data Bank (PDB) have been very useful in this regard. Herein, we aim to study three suspected Erg11p mutations such as Erg11pTHR285ALA, Erg11p_LEU321PHE, and Erg11p_SER457PRO for their involvement in fluconazole resistance using in-silico methods [9, 10].

## MATERIALS AND METHODS

**Selection of Erg11p residue mutations** **: **The point mutations within the *ERG11* gene that caused amino acid changes in Erg11 protein were selected based on previous reports as shown in Table 1. Three suspected mutations and a few experimentally verified mutations responsible for increased fluconazole minimum inhibitory concentration (MIC) were considered in this study [9-12].

**Figure 1:**
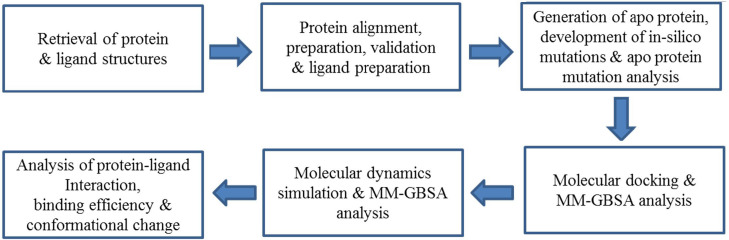
Schematic representation of the workflow.

**Workflow and computational components**: The workflow is shown as a flow chart (Fig. 1). All the computational works were done on Intel® Xeon® E5-2667 v3 Processor CPU @ 3.20 GHz with 16GB DDR4 RAM. Schrodinger 2017-1 and Desmond 2016-4 were compiled and run under Linux 16.04 LTS platform.

**Table 1:** Erg11 protein mutations reported in the fluconazole-resistant *Candida albicans*

**S.No**	**Residue Mutations**	**Verified**	**References**
1.	Erg11p_TYR132HIS	Yes	[12]
2.	Erg11p_THE285ALA	No	[10]
3.	Erg11p_LEU321PHE	No	[9]
4.	Erg11p_SER405PHE	Yes	[12]
5.	Erg11p_TYR447HIS	Yes	[11,12]
6.	Erg11p_SER457PRO	No	[10]
7.	Erg11p_GLY464SER	Yes	[12]
8.	Erg11p_ARG467LYS	Yes	[12]

**Retrieval of protein and ligand structures:** Crystal structures of C. albicans Erg11 protein (PDB ID: 5V5Z, monomer; 5FSA and 5TZ1, homodimer) were retrieved from the Protein Data Bank (PDB) [13]. 3D conformer of fluconazole (PubChem CID: 3365) was obtained from the PubChem database [14].

**Protein alignment, preparation, validation and ligand preparation:** Fasta format of the crystal structure sequences was downloaded and Clustal Omega multiple sequence alignment was used to align the structures with Erg11p sequence obtained from the Candida Genome Database (CGD) [15-17]. The obtained crystal structures contained co-crystal structures of ligands (azole drugs) and protoporphyrin IX (heme-containing small molecule). Maestro is a graphical user interface (GUI) of the Schrödinger suite. The protein preparation wizard in the Maestro (version 11.1.011) was used to prepare the protein structures by removing the ligand molecules and keeping protoporphyrin IX intact where heme plays a key role in Erg11p-drug interactions [18]. Thereafter, missing hydrogen atoms were filled. Furthermore, missing residues were cross-checked using the sequence viewer to ensure that they are included in the sequence of the imported structures. Subsequently, missing loops were filled with the “Prime” tool which builds an accurate loop model using the missing residues. Finally, protonation states were assigned and restrained minimization was done using the force field OPLS_2005 (Protein Preparation Wizard, Schrödinger, LLC, New York, NY, 2017-1) [19, 20]. Prepared protein structures were subjected to MDS for 50ns (50000ps) and validation was done by calculating the values of RMSD, RMSF and internal energy. Fluconazole structure was prepared using the LigPrep protocol. Before preparation, the structures were energy minimized using the OPLS_2005 force field and allowed to generate all possible biological ionization states at pH ranging 7.0 ± 2.0. However, it was not allowed to generate tautomer and options were set to retain chirality and allowed to generate one structure per ligand (LigPrep, Schrödinger, LLC, New York, NY, 2017-1) [21, 22].

**Generation of apo-protein, development of in-silico mutations and apo-protein mutation analysis:** Prepared protein structure (5V5Z) was used as the starting template for apo-protein (protein structure without ligand) generation by removing protoporphyrin IX. Incorporation of suspected mutations Erg11p_THE285ALA_apo, Erg11p_LEU321PHE_apo, Erg11p_SER457PRO_apo and a verified mutation Erg11p_TYR132HIS_apo were done using mutation tool [23]. Also, structures containing protoporphyrin IX were used to incorporate residue mutations such as Erg11p_TYR132HIS, Erg11p_THE285ALA, Erg11p_LEU321PHE, Erg11p_SER405PHE, Erg11p_TYR447HIS, Erg11p_GLY464SER, Erg11p_ARG467LYS and Erg11p_SER457PRO (three-letter code of amino acids were used throughout the text; mutated amino acids are underlined). The side-chain conformations were selected according to the best available conformation using the rotamer tool in the Schrödinger suite. All the mutant structures were subjected to a short minimization to fix the minor changes which occurred due to mutations (Protein Preparation Wizard, Schrödinger, LLC, New York, NY, 2017-1) [24]. The generated apo-protein and mutant apo-proteins were subjected to an extended 25ns MDS for analyzing the structural stability of the protein that occurred due to mutations. 

**Molecular docking: **The crystal structure 4WMZ of yeast *Saccharomyces cerevisiae* CYP51 protein contains fluconazole in its active site. The selected structure 5V5Z has a similar active site as that of 4WMZ; however, it contains itraconazole instead of fluconazole. The structures containing protoporphyrin IX with developed mutations were used in the docking study. Azole binding site of 5V5Z was used as a grid center for docking fluconazole with reference to 4WMZ. Water molecules were retained around 5Å near the hetero group due to their key role in Erg11p-fluconazole interactions [18]. Molecular docking was performed for fluconazole against all the Erg11p mutant structures generated and Erg11p_WT as well using extra precision (XP) docking method provided in the Glide (Grid-based Ligand Docking with Energetics) tool. Options were set for generating a maximum of 5000 poses per ligand for the initial phase of docking with a scoring window of 100.00 kcal/mol. This retains energetically favorable top 800 poses per ligand which were subjected to energy minimization and scoring. The docking used here is a flexible ligand sampling method with settings for sample nitrogen inversions and conformations that penalize nonplanar amides. The ligand conformations were evaluated using ChemScore function for energy-minimized poses that ranked the ligand poses by establishing a composite Emodel score where it rejects poses with Coulomb-vdW energy greater than 0.0 kcal/mol. Only distinct conformations are retained by using RMSD of less than 0.5 Å and maximum atoms displacement is less than 1.3 Å with strain energy corrections of 4.00 kcal/mol and 0.25 scaling factor for excess strain energy (Glide, Schrödinger, LLC, New York, NY, 2017-1). The consistency of ligand poses was verified by superposition of the top eight poses and calculating the RMSD values concerning the first pose. Images for docking were done using the Maestro interface (version 11.1.011) [25-27].

**Molecular dynamics simulations:** Molecular dynamics simulations were carried out using an explicit TIP4P water model to perform high-speed extensive simulation on biological systems using the Desmond package. Erg11p is a membrane protein, therefore, membrane placement was done using positions retrieved from the PDB of transmembrane proteins. The membrane setup using a POPC membrane model was built with the system building panel by assigning the periodic boundary condition of orthorhombic box size with a distance of 10 Å unit buffer. The system charge was balanced by adding sodium or chloride ions to neutralize the system and subsequently, apo-proteins and protein-ligand complexes were fixed appropriately in the solvated system. Then it was allowed to relax before simulation with a short minimization by default setting using OPLS_2005 force-field present in the Desmond suite. Further explicit MDS was carried out using the NPT ensemble at 300 K temperature and 1 atmospheric pressure using Nose-Hoover thermostat and Martyna-Tobias-Klein barostat scaling controls for a specified time of 50ns. Erg11p–fluconazole interaction percentage and fractions at the binding cavity were analyzed throughout the 50ns time. Visualization was done using the Maestro interface (version 11.0.014). Images for MD simulations were generated using simulation interaction diagram, event analysis and simulation quality analysis panel in the Desmond suite (Desmond, D. E. Shaw Research, New York, NY, 2016-4) [28-31].

**Molecular mechanics energies with generalized born surface area (MM-GBSA):** Binding free energy for Erg11p–fluconazole docked complexes were calculated using the Prime MM-GBSA tool in the Schrödinger suite which uses a novel energy generation model VSGB 2.0 (variable-dielectric surface generalized born model) for calculating the binding free energy (Prime, Schrödinger, LLC, New York, NY, 2017-1) [32, 33].

The equation to calculate binding free energy: ΔG (binding) = ΔG (complex) - (ΔG (free receptor) - ΔG (free ligand))

Where ΔG (binding) - binding free energy, ΔG (complex) - free energy of protein-ligand complex, ΔG (free receptor) - free energy of protein, ΔG (free ligand) - free energy of ligand.

## RESULTS

**Figure 2:**
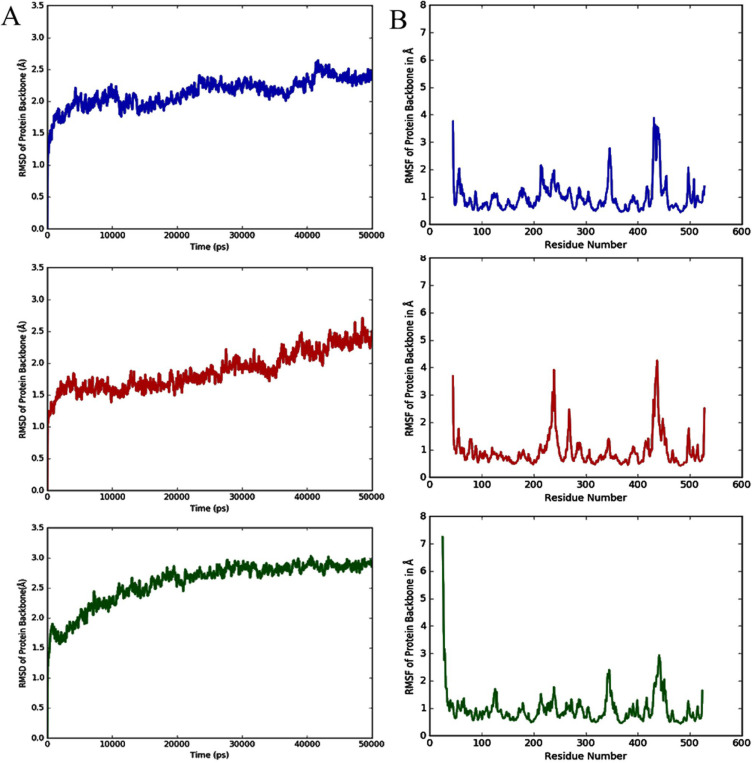
Molecular dynamics simulations analysis (50ns or 50000ps) for all the Erg11p crystal structures (A) RMSD plot, and (B) RMSF plot (Colour representation: Blue-5FSA, Red- 5TZ1 and Green-5V5Z)

Sequence alignment of 5FSA, 5TZ1 and 5V5Z with Erg11 protein sequence revealed that all the three structures obtained from the PDB lacked few residues due to crystallization error. 5FSA and 5TZ1 were similar to each other and lacked first 44 residues at N-terminus and had six mutations compared to wild type sequence. However, 5V5Z lacked the first 24 residues at N-terminus, the last 4 residues at C-terminus and 12 residues in the middle of the structure. N-terminal segments with missing residues are unusually long and cannot be filled. On the other hand, only 4 C-terminal residues are missing in 5V5Z and it has been considered as negligible. Internal missing residues in 5V5Z were filled by the “Prime” tool (an accurate protein structure prediction tool) at Schrödinger suite. The sequence present in 5V5Z is found to be more similar to wild type Erg11p than that of 5FSA and 5TZ1. Molecular dynamics simulations were performed for 50ns (50000ps) to assess the quality of crystal structures and revealed that internal energy was stable for all the three structures. However, the RMSD value of 5V5Z was found to be more stable whereas RMSF value for 5V5Z showed the least amino acid fluctuations compared to 5FSA and 5TZ1 (Fig. 2).

**Figure 3:**
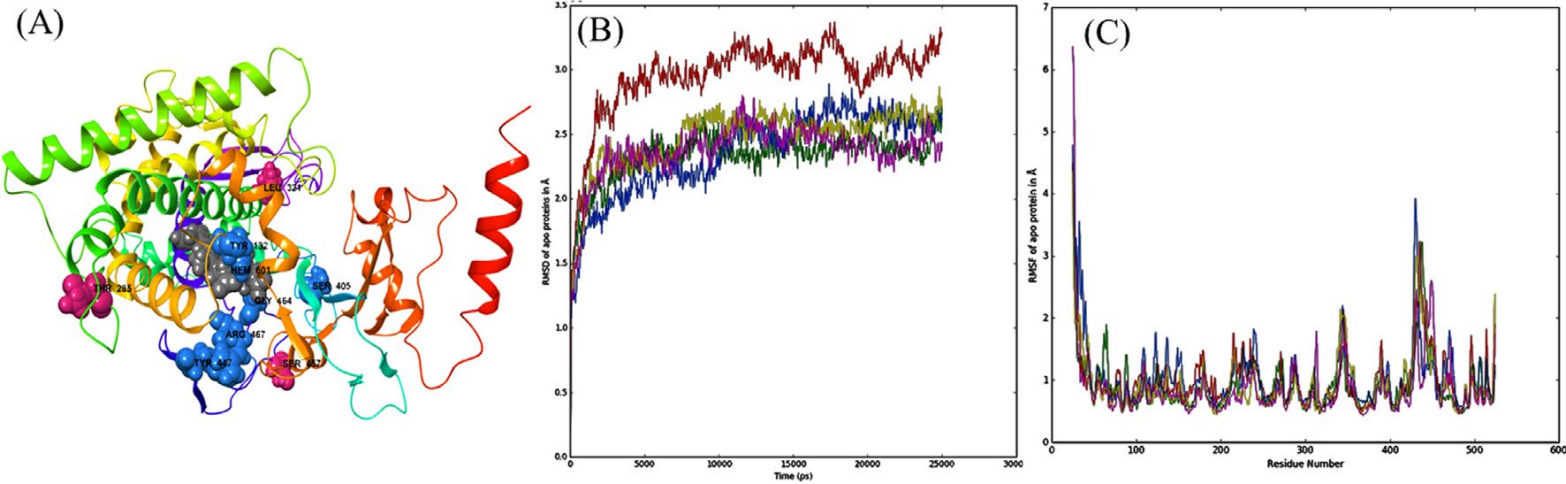
(A) Schematic of selected amino acid locations that have been mutated to TYR132HIS, THE285ALA, LEU321PHE, SER405PHE, TYR447HIS, SER457PRO, GLY464SER and ARG467LYS (verified fluconazole-resistant mutations – blue spheres; suspected mutations – pink spheres; heme – grey spheres). Apo-protein mutation analysis (B) RMSD plot, (C) RMSF plot (colour representation: green–Erg11p_WT_apo, red--Erg11p_TYR132HIS_apo, blue-Erg11p_THR285ALA_apo, yellow–Erg11p_LEU321PHE_apo and pink–Erg11p_SER457PRO_apo).

**Figure 4:**
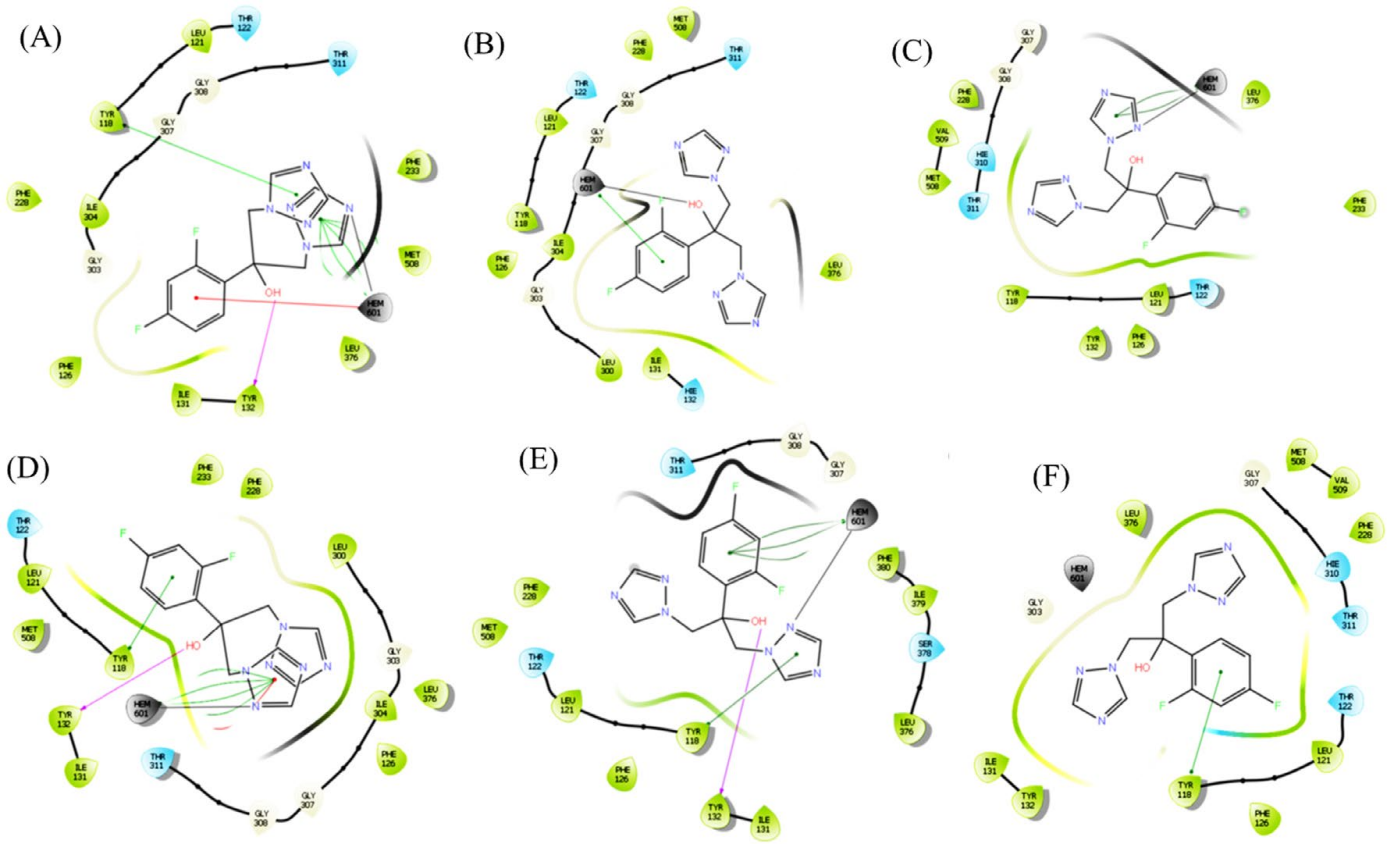
Two dimensional interaction pattern of Erg11p-fluconazole docked complexes (A) Erg11p_WT (B) Erg11p_TYR132HIS (C) Erg11p_TYR447HIS (D) Erg11p_THR285ALA (E) Erg11p_LEU321PHE (F) Erg11p_SER457PRO (fluconazole resides in the middle, surrounded by the residues. Residues & interaction types: light green spheres – hydrophobic; light blue spheres – polar; grey spheres – heme; white spheres – glycine; purple arrows – backbone hydrogen bonds; dotted purple arrows – side chain hydrogen bonds; green lines – Pi-pi stacking; red lines – Pi-pi cation; black lines – metal coordination).

**Figure 5:**
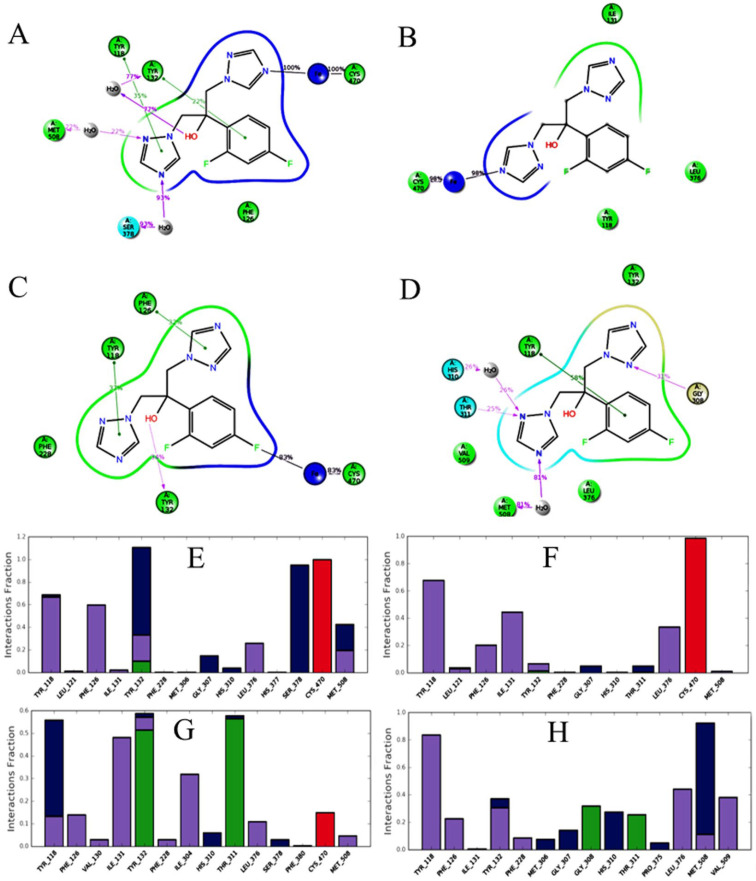
Erg11p-fluconazole interaction pattern after the MDS and the percentage of interaction (>10%) (A) Erg11p_WT, (B) Erg11p_TYR447HIS, (C) Erg11p_LEU321PHE, (D) Erg11p_SER457PRO (fluconazole resides in the middle, surrounded by the residues. Residues & interaction types: green sphere – hydrophobic; grey sphere – water; light blue – polar; dark blue – heme; light gold – glycine; purple arrows – backbone hydrogen bonds; dotted purple arrows – side chain hydrogen bonds; green lines – Pi-pi stacking; black lines – metal coordination). Histogram for the interaction fraction of residues that stabilize Erg11p-fluconazole docked complexes (E) Erg11p_WT (F) Erg11p_TYR447HIS (G) Erg11p_LEU321PHE (H) Erg11p_SER457PRO (interaction types: green bars – hydrogen bonds; grey bars – hydrophobic interactions; pink bars – metal coordination; dark blue bars – water bridges).

**Figure 6:**
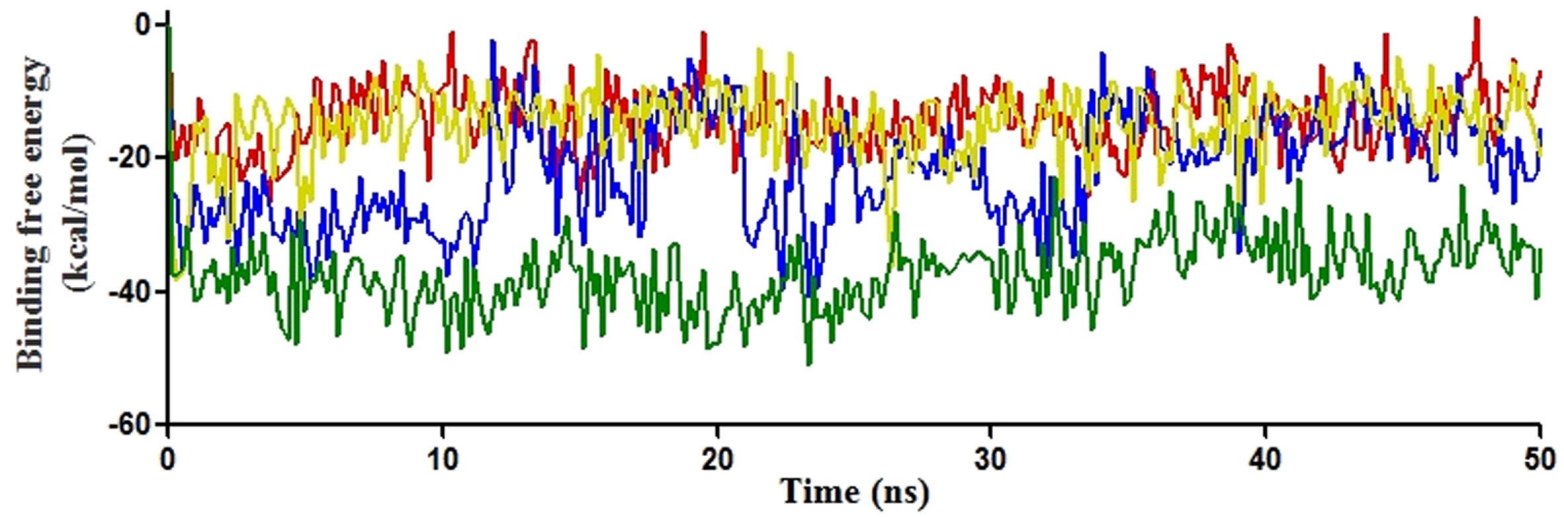
MM-GBSA binding free energy values throughout the 50ns MDS for Erg11-fluconazole docked complexes (colour representation: green - Erg11p_WILD_TYPE; yellow - Erg11p_TYR447HIS; blue - Erg11p_LEU321PHE; red - Erg11p_SER457PRO).

**Schematic of eight wild type residue locations in the Erg11p crystal structure and its respective residue mutations are shown in** Figure 3A **. Individual mutations developed using the selected Erg11p crystal structure (5V5Z) containing heme is shown in Supplement** Figure 1 **.**

The RMSD and RMSF analyses for wild type apo-protein, suspected mutations (Erg11p_THR285ALA_apo, Erg11p_VAL321LEU_apo, Erg11p_SER457PRO_apo) and verified mutation Erg11p_TYR132HIS_apo are shown in Figure 3B-C.

Apo-protein analysis revealed that RMSD values of suspected mutations were similar to that of wild type Erg11p and lesser than that of Erg11p_TYR132HIS_apo. However, there is no much variation in the RMSF values among the mutations that were analyzed.

**The developed Erg11p mutant structures along with wild type Erg11p were subjected to molecular docking against antifungal drug fluconazole. The superimposition of the top eight fluconazole poses and RMSD calculations concerning the first frame showed that fluconazole had a stable pose with less deviation. This confirmed the consistency of the docking pose (Supplement** Fig. 2 **and Supplement** Table 1 **). Thereafter, the ranking was done based on non-bonded interaction, Glide Emodel score and Gibbs binding free energy (** Fig. 4 **and** Table 2 **). The Erg11p-fluconazole docking interaction results for (A) Erg11p_WT (B) Erg11p_TYR132HIS (C) Erg11p_TYR447HIS (D)** **Erg11p_THR285ALA (E) Erg11p_LEU321PHE and (F) Erg11p_SER457PRO are shown in **Figure 4**. All other Erg11p mutant-fluconazole interactions are shown in supplement **Figure 3**.**

Glide Emodel score for the best pose selection showed a greater binding affinity for Erg11p_WT compared to other mutants. Erg11p_WT showed the least value of -65.436 kcal/mol and the verified mutation Erg11p_TYR132HIS showed a higher value of -58.676 kcal/mol. MM-GBSA binding free energy analysis also showed the least value for Erg11p_WT compared to other mutants except for Erg11p_THR285ALA. The binding free energy value for Erg11_WT was -54.33 kcal/mol (the lowest value) whereas -35.04 kcal/mol for the mutant Erg11p_TYR447HIS is the highest.

**Table 2:** Predicted glide emodel score and MM-GBSA score for the fluconazole docked with Erg11p wild type and mutants

**S.No**	**Erg11p wild** **type and mutants docked ** **with fluconazole**	**Glide emodel Score** **(kcal/mol)**	**MM-GBSA score ** **(kcal/mol)**
1	Erg11p_TYR447HIS	-58.756	**-35.04**
2	Erg11p_SER457PRO	-62.214	**-36.12**
3	Erg11p_ARG467LYS	-59.504	**-37.39**
4	Erg11p_SER405PHE	-60.823	**-40.23**
5	Erg11p_LEU321PHE	-60.177	**-43.11**
6	Erg11p_GLY464SER	-61.343	**-43.36**
7	Erg11p_TYR132HIS	-58.676	**-51.88**
8	Erg11p_WT	-65.436	**-54.33**
9	Erg11p_THE285ALA	-62.650	**-60.78**

Docking and MM-GBSA results suggested that Erg11_LEU321PHE and Erg11p_SER457PRO might involve in fluconazole resistance. Therefore, Erg11p_SER457PRO-fluconazole and Erg11_LEU321PHE-fluconazole docked complexes were subjected to explicit molecular dynamics simulation for an extended 50ns. Similar analyses were done for wild type Erg11p and a verified mutation Erg11p_TYR447HIS that gave the least binding free energy and reduced interactions with fluconazole. After the molecular dynamics simulations Erg11p–fluconazole interactions were analyzed.

After 50ns molecular dynamics simulations, it has been found that wild type Erg11p-fluconazole interactions had the highest number of contacts (TYR_118, PHE_126, TYR_132, GLY_307, LEU_376, SER_378, CYS_470, MET_508 & HEME) and higher percentage of interactions as well. However, the verified mutation Erg11p_TYR447HIS showed reduced residue contacts (TYR_118, PHE_126, ILE_131, LEU_376, CYS_470 & HEME) and lower percentage of interactions. On the other hand, suspected mutation Erg11p_LEU321PHE-fluconazole interaction had shown contacts with TYR_118, PHE_126, TYR_132, ILE_131, THR_311, ILE_304, LEU_376, CYS_470 & HEME whereas suspected mutation Erg11p_SER457PRO-fluconazole interaction had contacts with TYR_118, PHE_126, TYR_132, GLY_307, GLY_308, HIS_310, HIS_311, LEU_376, MET_508, VAL_509 & HEME. This showed that the number of residue contacts of fluconazole with wild type and suspected mutations is more or less the same but the percentage of interaction was less. Moreover, for suspected mutations, fluconazole had interacted with few different residues than that of wild type Erg11p (Fig. 5).

The histogram for interaction of fluconazole with specific residues showed that the fraction of participating residues is ≥ 0.4 for Erg11p_WT (TYR_118, PHE_126, TYR_132, SER_378, CYS_470, MET_508), Erg11p_TYR447HIS (TYR_118, ILE_131, CYS_470), Erg11p_LEU321PHE (TYR_118, ILE_131, TYR_132, THR_311) and Erg11p_SER457PRO (TYR_118, LEU_376, MET_508). There are at least five different mode of interactions through which Erg11p and fluconazole complexes are stabilized. These are for Erg11p_WT( hydrophobic interaction**-**TYR_118, PHE_126, TYR_132, MET_508; water bridges–TYR_118, TYR_132, SER_378, MET_508; hydrogen bond–TYR_132; Pi-pi stacking–TYR_118, TYR_132; metal coordination–CYS_470, HEME); Erg11p_TYR447HIS (hydrophobic interaction - TYR_118; pi stacking**–**PHE_126; metal coordination–CYS_470, HEME); Erg11p_LEU321PHE (hydrophobic interaction-TYR_118, ILE_131, TYR_132; water bridges-TYR_118, TYR_132; hydrogen bond–TYR_132, THR_311; Pi-pi stacking–TYR_118); Erg11p_SER457PRO (hydrophobic interaction-TYR_118, LEU_376, MET_508; water bridges – MET_508; Pi-pi stacking – TYR_118) (Fig. 5E-H). 

MDS data of 50ns after docking were used to calculate MM-GBSA binding free energy. Subsequently, it was used to comprehend the structural decomposition of Erg11p-fluconazole complexes such as Erg11p_WT, Erg11p_TYR447HIS, Erg11p_LEU321PHE and Erg11p_SER457PRO (Fig. 6 and Supplement Table 2).

The MM-GBSA binding free energy values calculated throughout 50ns MDS produced a wide range of values. Erg11p_WT showed the least binding free energy value of -50.886 kcal/mol and the highest value of -22.648 kcal/mol. On the other hand, mutants showed lower and higher values of -38.171 kcal/mol and -3.486 kcal/mol for Erg11p_TYR447HIS, -40.853 and -4.1796 for Erg11p_LEU321PHE and -26.785 kcal/mol and 1.208 kcal/mol for Erg11p_SER457PRO.

## DISCUSSION

In-silico techniques have been extremely useful in identifying the crucial mutations that may involve in drug resistance. In this study, we chose three suspected mutations in Erg11 protein that have been reported in fluconazole-resistant clinical isolates of *C. albicans*. The first and foremost step for the in-silico study is to select the best crystal structure. The appropriate template selection was done by aligning all the three available crystal structure sequences of *C. albicans* with wild type sequence of Erg11p (Fig. 2A). MDS analysis for 50ns (50000ps) revealed that the structure 5V5Z had stable internal energy, consistent RMSD value and least RMSF value (Fig. 2B-C). All these results showed that 5V5Z is the best template out of the three Erg11p crystal structures 5FSA, 5TZ1 and 5V5Z [13]. 

The selected structure was used to depict the residues and their respective mutations (Fig. 3A and Table 1) [9-12]. The in-silico study was started with Erg11 apo-protein (without co-crystal structures) to know the structural changes or deformation due to the suspected mutations. In this study, all three suspected mutations as well as wild type Erg11p (negative control) and a verified mutation Erg11p_TYR132HIS (positive control) were developed using apo-protein [23]. Developed apo-proteins were subjected to 25ns MDS to have insights into the conformational stability of the proteins due to mutations. MDS of apo-proteins for the suspected mutations showed little variation in the RMSD value compared to wild type but verified mutation Erg11p_TYR132HIS_apo showed a higher value as expected (Fig. 3B). There is no significant change in the RMSF value for any of the mutations compared to wild type (Fig. 3C). This indicates that there is only a little change in the tertiary structure of the protein due to the suspected mutations. Therefore, resistance caused by the suspected mutations could occur due to the inefficient binding of fluconazole to the enzyme or change in the conformation at the binding cavity. 

Binding study of fluconazole was performed by extra precision (an accurate flexible docking method that avoids false-positive results) to assess the binding of fluconazole with mutated Erg11 proteins [26]. Superimposition of the top eight fluconazole poses for all the docked complexes clearly showed that the docking pose of fluconazole was consistent with lesser deviations (Supplement Fig. 2 and Supplement Table 1). Docking was followed by MM-GBSA analysis to determine the stability of the binding efficiency. The docking and MM-GBSA results showed that mutated Erg11p had reduced non-bonded interactions and higher binding free energy compared to wild type Erg11p. The interaction of fluconazole with suspected mutation Erg11p_THR285ALA is found to be similar to wild type Erg11p and showed the least binding free energy value. However, Erg11p_LEU321PHE had lesser interaction with fluconazole and higher binding free energy than that of Erg11p_THR285ALA but lesser than Erg11p_SER457PRO. The mutation Erg11p_SER457PRO showed reduced non-bonded interaction and higher binding free energy value with fluconazole compared to all other suspected mutations and the second highest among all the verified mutations. In agreement with the experimental results, all the verified mutations showed reduced non-bonded interactions and higher binding free energy values than that of wild type Erg11p. Among the verified mutations, Erg11p_TYR447HIS showed the highest binding free energy and reduced non-bonded interactions (Fig. 4 & Table 2). This finding revealed that two suspected mutations Erg11p_LEU321PHE and Erg11p_SER457PRO could be involved in fluconazole resistance. 

Previous studies reported that water-mediated interactions of Erg11p residues TYR_126, TYR_140 and SER_382 of *S. cerevisiae *play an important role in fluconazole resistance [18]. In *C. albicans* Erg11p, TYR_118, TYR_132 and SER_378 are the corresponding positions of those amino acids. To analyze the mode of Erg11p-fluconazole interactions and stability, 50ns MDS was performed for two suspected mutations (Erg11p_LEU321PHE and Erg11p_SER457PRO) complexed with fluconazole whereas verified mutation Erg11p_TYR447HIS and wild type Erg11p serve as positive and negative controls, respectively.

MDS results revealed that Erg11p of *C. albicans* had similar water-mediated interactions with fluconazole involving TYR_132 and SER_378 residues. Furthermore, TYR_118 had shown higher interaction with fluconazole, however, it lacks hydrogen or water-mediated hydrogen bonds. This implies that TYR_132 and SER_378 might have a critical role in drug resistance of *C. albicans* as reported in *S.* *cerevisiae* [18]. TYR_118 could also play a critical role in the interaction with fluconazole. Interestingly, these water-mediated interactions are completely lacking in verified mutation Erg11p_TYR447HIS. However, Erg11p_LEU321PHE is the only mutation that showed strong water-mediated interaction for TYR_118, a non-water mediated hydrogen bond for TYR_132 and weak water-mediated hydrogen bonds for TYR_132 and SER_378 residues. However, Erg11p_SER457PRO showed a very little water-mediated hydrogen bond interaction for TYR_132. Wild type Erg11p and verified mutation Erg11p_TYR447HIS showed strong interaction with heme. However, suspected mutation Erg11p_LEU321PHE showed weak heme interaction whereas Erg11p_SER457PRO mutant completely lacked heme interaction (Fig. 5A-H). This suggests that Erg11p_LEU321PHE mutation could have reduced binding efficiency with fluconazole due to weak non-bonded and heme interactions. However, Erg11p_SER457PRO mutation lacked crucial water-mediated and heme interactions that may lead to the least binding efficiency with fluconazole. 

MM-GBSA analysis was performed followed by MDS to understand the binding efficiency of fluconazole to Erg11p. These analyses showed the least binding free energy for wild type Erg11p throughout the simulation and suspected mutation Erg11p_LEU321PHE comes next to wild type. However, Erg11p_SER457PRO showed higher binding free energy similar to that of a verified mutation Erg11p_TYR447HIS (Fig.6, Supplement Table 2). These results revealed that the interaction pattern of fluconazole with Erg11p_THR285ALA is closely matching with wild type Erg11p. The binding free energy was also lesser than the wild type Erg11p. This indicates that Erg11p_THR285ALA mutation may not have any significant role in fluconazole resistance. Erg11p_LEU321PHE-fluconazole docking showed fewer non-bonded interactions with fluconazole and increased binding free energy compared to wild type Erg11p. On the other hand, MDS showed a reduced water-mediated hydrogen bond and heme interactions for this mutant. However, MM-GBSA analysis of post-MDS produced lesser binding free energy for Erg11p_LEU321PHE mutation compared to suspected mutation Erg11p_SER457PRO and verified mutation Erg11p_TYR447HIS. These analyses indicate that Erg11p_LEU321PHE mutation might have a moderate role in fluconazole resistance as reported by Carvalho et al., 2013 [9]. However, Erg11p_SER457PRO is the only suspected mutation that showed very less non-bonded interactions and higher binding free energy value compared to wild type Erg11p. Moreover, it lacked the important heme interaction that involves in the Erg11p-fluconazole binding. MM-GBSA analyses of post-MDS produced the highest binding free energy for Erg11p_SER457PRO mutation. This confirms that this mutation caused a significant reduction in the binding efficiency of fluconazole to Erg11 protein.

Our study strongly suggests that Erg11p_SER457PRO mutation may play an active role in fluconazole resistance. However, suspected mutation Erg11p_LEU321PHE could have a moderate role whereas Erg11p_THR285ALA may not have any significant role in fluconazole resistance. Further validation using the experimental methods will confirm the involvement of these mutations in fluconazole resistance. Moreover, this study will help in exploring other suspected drug-resistant mutations of Erg11 protein. This could pave the way for developing new versions of antifungals to overcome the growing drug resistance in fungal pathogen *C. albicans*. 
